# The declarative system in children with specific language impairment: a comparison of meaningful and meaningless auditory-visual paired associate learning

**DOI:** 10.1186/s40359-015-0062-7

**Published:** 2015-02-19

**Authors:** Dorothy V M Bishop, Hsinjen Julie Hsu

**Affiliations:** Department of Experimental Psychology, University of Oxford, Tinbergen Building, South Parks Road, OX1 3UD Oxford, UK; Current address: Graduate Institute of Audiology and Speech Therapy, National Kaohsiung Normal University, Kaohsiung, Taiwan

**Keywords:** Specific language impairment, Learning, Procedural deficit hypothesis, Declarative, Procedural, Vocabulary, Training, Memory

## Abstract

**Background:**

It has been proposed that children with Specific Language Impairment (SLI) have a selective deficit in procedural learning, with relatively spared declarative learning. In previous studies we and others confirmed deficits in procedural learning of sequences, using both verbal and nonverbal materials. Here we studied the same children using a task that implicates the declarative system, auditory-visual paired associate learning. There were parallel tasks for verbal materials (vocabulary learning) and nonverbal materials (meaningless patterns and sounds).

**Methods:**

Participants were 28 children with SLI aged 7–11 years, 28 younger typically-developing children matched for raw scores on a test of receptive grammar, and 20 typically-developing children matched on chronological age. Children were given four sessions of paired-associate training using a computer game adopting an errorless learning procedure, during which they had to select a picture from an array of four to match a heard stimulus. In each session they did both vocabulary training, where the items were eight names and pictures of rare animals, and nonverbal training, where stimuli were eight visual patterns paired with complex nonverbal sounds. A total of 96 trials of each type was presented over four days.

**Results:**

In all groups, accuracy improved across the four sessions for both types of material. For the vocabulary task, the age-matched control group outperformed the other two groups in the starting level of performance, whereas for the nonverbal paired-associate task, there were no reliable differences between groups. In both tasks, rate of learning was comparable for all three groups.

**Conclusions:**

These results are consistent with the Procedural Deficit Hypothesis of SLI, in finding spared declarative learning on a nonverbal auditory-visual paired associate task. On the verbal version of the task, the SLI group had a deficit in learning relative to age-matched controls, which was evident on the first block in the first session. However, the subsequent rate of learning was consistent across all three groups. Problems in vocabulary learning in SLI could reflect the procedural demands of remembering novel phonological strings; declarative learning of crossmodal links between auditory and visual information appears to be intact.

## Background

In a series of papers, Ullman and colleagues made the case that vocabulary and grammar predominantly engage different neural systems (Ullman 2001, 2004; Ullman et al. 1997). A fundamental distinction was drawn between the mental lexicon, a repository of information about phonological forms and their associated meanings, and the grammatical system, which computes the meanings of complex forms using the rules of grammar. According to the declarative/procedural model of language, these two kinds of processing are most efficiently handled by different systems: the declarative system for the lexicon and the procedural system for grammar. These memory systems are not specific to language, and have been studied in various animal models, especially monkeys and rodents (see Eichenbaum 2002 for review). They have been termed as systems for ‘knowing that’ (declarative) versus ‘knowing how’ (procedural) (Squire 1982). Ullman and Pierpont (2005) went on to argue that specific language impairment (SLI), a condition where language learning lags behind other aspects of development, involves a selective impairment of the procedural memory system, with relative preservation of declarative memory. This should lead to disproportionate difficulties with grammatical development, and where children do learn, they may rely heavily on rote learning, mediated by the declarative system, rather than abstraction of general grammatical rules.

Procedural memory is involved in learning of new skills, including motor skills such as riding a bicycle. Early evidence for a distinction between declarative and procedural memory came from a demonstration by Milner (1970) that the densely amnesic patient HM could learn a hand–eye coordination skill (mirror drawing) despite having no memory of having done the task before. A key feature of procedural memory is that it is implicit, i.e., the knowledge of what has been learned is not available to introspection. This fits well with what we know about grammatical knowledge; people who produce language fluently typically cannot explain the grammatical rules they use. The procedural memory system involves circuits in the frontal lobes and basal ganglia. Ullman (2004) argued that there are parallel circuits in the basal ganglia which conduct similar computations but over different domains, in particular motor sequence learning and grammatical rules. Broca’s area, which is a key component of the procedural memory system, is thought to be implicated in learning abstract sequences that are hierarchically structured. However, it would be misleading to imply too neat a neuroanatomical division between procedural and declarative systems; for instance, Broca’s area is also involved in some aspects of declarative memory, notably selection of lexical items (Heim et al. 2009).

Initial accounts of the declarative system emphasized its role in learning facts and specific episodes that can be explicitly recalled. Early work on this memory system relied on evidence from individuals who could no longer remember facts or experiences after brain damage. Such cases of amnesia typically involve medial temporal lobe structures, especially the hippocampus. It has been proposed that these medial temporal lobe structures are needed to bind together information from different cortical regions (Squire and Zola-Morgan 1991).

The Procedural Deficit Hypothesis of Ullman and Pierpont (2005) makes two key predictions about learning in SLI. First, deficits should be observed in procedural learning, for non-language as well as language tasks. Second, declarative learning should be relatively spared. In support of the hypothesis, there is a growing body of work showing deficiencies in SLI, for both language and motor procedural tasks that involve learning of complex sequences (Lum et al. 2014 (meta-analysis); Hsu and Bishop 2014a). Nevertheless, children with SLI appear unimpaired on other tasks that are thought to be mediated by the procedural system, including pursuit rotor learning (Hsu and Bishop 2014a) or eyeblink conditioning (Hardiman et al. 2013). We have interpreted such findings by suggesting that there is a procedural deficit in SLI, but it is restricted to tasks that involve sequencing of discrete motor or verbal elements. Lee and Tomblin (2014), however, found that adults with persisting language impairment were impaired on pursuit rotor learning. It may be that there may be more widespread procedural impairment that is hard to demonstrate in children because their performance is more variable. There is also conflicting data on studies using a nonsequential ‘weather prediction’ probabilistic learning task (Kemény and Lukács 2010; Lee and Tomblin 2014; Lukács and Kemény 2014). However, it should be noted that this task is often solved using explicit strategies (Gluck et al. 2002) and its designation as an measure of procedural learning has been challenged (Newell et al. 2007). This illustrates that although the procedural/declarative distinction may appear clearcut in theory, in practice it can be hard to tease apart the two systems and devise a task that is a pure measure of just one of them.

Distinguishing procedural and declarative learning is complicated in the case of lexical knowledge. Vocabulary learning– generally regarded as involving declarative memory - is impaired in many children with SLI (Gray 2004, 2005; Gray et al. 1999; Watkins et al. 1995; Rice et al. 1992; Rice et al. 1990; Rice et al. 1994). Ullman and Pierpont (2005) argue, however, that this makes sense because vocabulary acquisition involves both procedural and declarative systems. In addition to motor and grammatical skills, the brain structures that constitute the procedural system are involved in other functions, some of which are relevant to vocabulary learning, such as word retrieval and working memory. It follows, therefore, that the extent to which vocabulary learning in SLI is impaired will depend on how this is assessed. Ullman and Pierpont argue that children with SLI are particularly likely to show deficits in tasks that involve word retrieval, rapid presentation of stimuli or high demands on working memory.

One prediction from this account is that children with SLI should perform on vocabulary tasks like typically-developing controls who are matched on procedural skills (e.g., grammatical ability). A second prediction is that relatively good performance on vocabulary learning should be found if demands on functions that draw on the procedural system are reduced. As Nation (2014) noted, learning a new word involves many different processes, including the ability to use syntactic bootstrapping to infer meaning from grammatical context, and phonological segmentation and memory. Weak phonological short-term memory is one of the most robust and consistent findings in SLI (Graf Estes et al. 2007), and vocabulary learning will be impacted by this, especially at the initial stage of learning. Relatively good performance in children with SLI should be found if demands on phonological short-term memory are reduced and contextual cues from syntax and other sources are excluded: as Ullman and Pierpont (2005) argued: “Word learning should be quite easy when items are presented slowly and in a rich semantic context, facilitating memorization in declarative memory” (p. 418). Consistent with this, Lum and Conti-Ramsden (2013) reviewed the literature on this topic and concluded that although verbal declarative memory appeared impaired in SLI, this was because initial learning was affected by poor working memory. If this was controlled for, then there was less evidence of deficits. Furthermore, there was no evidence of a declarative deficit on nonverbal tasks. Additional evidence came from a study of novel word learning in adults with SLI (McGregor et al. 2013). These authors found impairments in encoding of phonological forms, but the SLI group was not specifically impaired in linking word forms to meaning and remembering these links.

The current study was designed to evaluate declarative learning in SLI. It incorporated a number of features that build on and extend prior studies. First, we used a vocabulary learning task that adopted an auditory-visual paired-associate method. This is different from the tasks reviewed by Lum and Conti-Ramsden (2013), which involved learning of word pairs or word lists composed from existing vocabulary. Our task was closer to the kind of novel word-learning task used by McGregor et al. (2013), in that it involved combining information from different modalities to form a new vocabulary item in memory, linking phonology and meaning. Second, we attempted to minimize the role of phonological short-term memory on performance: the task did not require any speech production, and learning was assessed by having the child select the correct picture to match a spoken form. The spoken forms were selected to be distinctive. Third, we looked at learning over four sessions on different days. This meant that we could consider retention and consolidation of learned information over time as well as within-session learning. Fourth, we gave children a nonverbal paired-associate learning task that used an identical format, so we could directly compare verbal and nonverbal declarative learning. As far as we are aware, this has not previously been done. Finally, we compared children with SLI with two groups: age-matched controls and grammar-matched controls. The latter were children who were two to three years younger than those with SLI, but who performed similarly on a test of receptive grammar. This allowed us to see whether any learning deficits in those with SLI were in line with immature language skills, or whether they were atypical for any age. Following the reasoning of Lum and Conti-Ramsden (2013), we would expect any deficits of children with SLI in verbal declarative learning to disappear when compared to children whose verbal memory was similar.

We made the following predictions, based on the Procedural Deficit Hypothesis, and on the review of existing work by Lum and Conti-Ramsden (2013):

1. Relative to age-matched controls, children with SLI will be impaired at initial learning on a verbal paired-associate task, but their rate of learning will be normal.
2. Performance on verbal paired-associate learning by children with SLI will be comparable to that of younger children matched on grammatical comprehension.
3. On a nonverbal paired-associate learning task in which no encoding of phonological forms or remembering novel phonological strings is required, children with SLI will be unimpaired relative to age-matched controls.
4. Performance on verbal paired-associate learning will be predictable from a measure of verbal short-term memory, and any differences from age-matched controls will be diminished or abolished when this is taken into account.

## Methods

### Ethics approval

Approval for this study was given by the University of Oxford Medical Sciences Division Research Ethics Committee, approval reference MSD/IDREC/2009/28. Parents of all participants gave written informed consent, and the children gave assent after the study was explained in age-appropriate language.

### Data and material release

Raw data from this project are available on http://dx.doi.org/10.6084/m9.figshare.1292889. Analysis scripts and other materials are available on the Open Science Framework: https://osf.io/bwnph/?view_only=035c7791e5564d2598da61e000c66bad.

### Participants

Children taking part in this study were a subset of those described in our previous reports on nonverbal procedural learning (Hsu and Bishop 2014a) and training of sentence comprehension (Hsu and Bishop 2014b). We studied three groups of children: a) 7 to 11 year-old children with SLI (N =28); (b) typically-developing children matched on chronological age (Age-matched, N =20); and (c) younger typically-developing children matched for raw scores on a test of receptive grammar (Grammar-matched, N = 28).

The children with SLI were recruited from special schools for children with language impairment or support units in mainstream schools. Children were included if they met all of the following screening criteria:

1. Score at least one SD below the mean on at least two out of the following six standardized tests: the British Picture Vocabulary Scales II, BPVS II, (Dunn et al. 1997), Test for Reception of Grammar-Electronic, TROG-E (Bishop 2005) the comprehension subtest of the Expression, Reception and Recall of Narrative Instrument, ERRNI, (Bishop 2004), repetition of nonsense words subtest of the Developmental Neuropsychological Assessment, NEPSY, (Korkman et al. 1998) and syntactic formulation and naming subtests of the Assessment of Comprehension and Expression 6–11, ACE 6–11 (Adams et al. 2001).
2. Nonverbal ability standard score of 85 or above, as measured with the Raven’s Coloured Progressive Matrices (Raven et al. 1986)
3. Able to hear a pure tone of 20 dB or less in the better ear, at 500, 1000, 2000 and 4000 Hz;
4. English as the native language;
5. Did not have a diagnosis of another developmental disorder such as autism, Down Syndrome or Williams Syndrome.

The same screening tests were used to confirm language status for each child in the grammar- and age-matched groups. These children met the same criteria for nonverbal ability, hearing and native language and did not have a z-score less than −1.0 on more than one of the six standardized language tests or have a history of speech, language, social or psychological impairments. Descriptive information on the participants is given in Table 1.

**Table 1:** Mean (SD) age and test scores for three groups*

	**SLI N = 28**	**Grammar-matched N = 28**	**Age-matched N = 20**
Age (yr)	8.6 (1.32)	5.8 (0.86)	8.9 (0.77)
Raven’s Matrices SS	102.9 (13.25)	105.2 (8.47)	105.8 (11.35)
TROG-E raw blocks	8.3 (4.00)^a^	9.8 (3.11)^a^	14.8 (2.38)^b^
TROG-E SS	73.3 (14.29)^a^	102.3 (13.96)^b^	97.8 (10.31)^b^
BPVSII raw	69.5 (17.77)^a^	67.1 (12.95)^a^	92.6 (9.16)^b^
BPVSII SS	87.8 (13.18)^a^	108.3 (9.51)^b^	102.6 (7.58)^b^
NEPSY nonwords raw	22.3 (8.90) ^a^	27.0 (7.60)	32.2 (8.48)^b^
NEPSY nonwords SS	84.1 (16.78)^a^	104.3 (15.01)^b^	101.8 (15.75)^b^
ERRNI Comprehension raw	8.3 (3.25)^a^	8.4 (3.12)^a^	12.9 (2.56)^b^
ERRNI Comprehension SS	82.5 (15.17)^a^	103.0 (14.6)^b^	101.9 (12.58)^b^
ACE Naming raw	10.2 (4.32)^a^	10.0 (2.84)^a^	16.3 (3.01)^b^
ACE Naming SS	81.6 (13.2)^a^	101.1 (7.98)^b^	98.8 (10.99)^b^
ACE Syntax raw	15.0 (5.16)^a^	17.8 (5.93)^a^	24.1 (5.13)^b^
ACE Syntax SS	80.7 (9.2)^a^	105.5 (14.16)^b^	98.5 (15.05)^b^
N impaired tests	3.3 (1.38)^a^	0.3 (0.48)^b^	0.4 (0.5)^b^
Word Span	4.0 (1.07)^a^	4.1 (0.88)^a^	4.9 (0.79)^b^

*Means with different superscripts differ significantly from one another on post hoc Sidak test, p < .05.

The children in the grammar-matched group were aged between 4 and 6 years and were matched individually with the children in the SLI group on TROG-E raw scores (i.e., number of blocks passed). Each child in the grammar-matched group had a TROG-E raw score within three blocks of one of the children in the SLI group. Group differences on TROG-E raw score were not significant between the SLI and the grammar-matched group. Furthermore, the grammar-matched group had similar raw scores to the SLI group on all other language measures except nonword repetition, where the grammar-matched group had significantly higher scores than the SLI group (see Table 1). In addition, these two groups showed equivalent performance on a nonverbal procedural learning task (Hsu and Bishop 2014a).

### Testing schedule

All children were seen on seven days during a two week period, during which they completed two screening sessions (language, hearing, nonverbal IQ), followed by four sessions of language training and a post-test session. In the four training sessions, each lasting 15–20 mins, children were given verbal and nonverbal auditory-visual paired associate tasks (see below) and training on comprehension of sentences where word order determines meaning (Hsu and Bishop 2014b).

### Vocabulary learning task

A computerised vocabulary learning task was devised for this study. The computer code to run the program is available on: osf.io/xrmjk/. Children learned a set of eight rare animal names (ayeaye, saki, dugong, anole, caiman, iiwi, kyloe, jennet). We trained understanding of real words, because we felt it would be unethical to have language-impaired children spend significant amounts of time learning meaningless materials. This limited the experimental control we had over word forms, but all the animal names were distinctive, low-frequency, bisyllabic words, of between 650 to 1000 ms duration. The same eight words were repeated in a pseudorandom order three times across a training session, but with different foils, as a measure of vocabulary learning. The same task was conducted for each training session.

**Figure 1:**
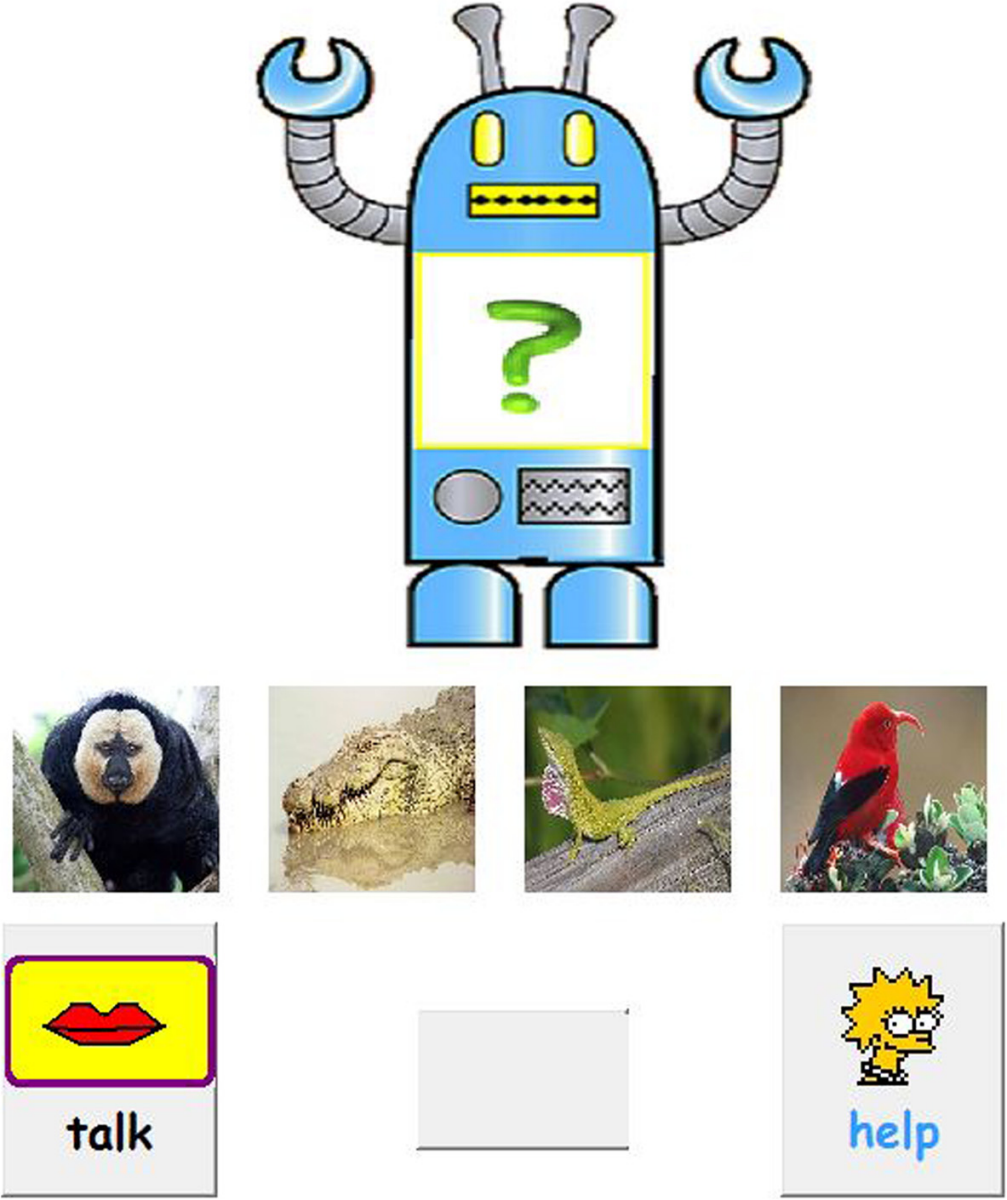
**Screenshot of vocabulary training game.**

On training session 1 only, children saw pictures of all eight items while the animals were named for them twice before the training began. To make sure children understood the task, two warm-up trials using familiar vocabulary were provided before training session 1. Each training session contained three blocks of the eight animal names, for a total of 24 training trials. On each trial, children heard a target word and saw an array of four pictures: one target picture and three foils (see Figure 1). They had to select the picture that matched the spoken name by clicking on the picture. Once a picture was clicked, it automatically moved inside a picture of a robot, located above the 4-picture array. If the child’s response was correct, the robot said the target word and the program moved to the next trial automatically. If the child’s response was incorrect, the selected picture still moved to the robot, but this time the robot did not say the target word. The child was then told by the examiner: “*The robot didn’t say the word. That must not be the right picture. Do you want to try another picture? Or you can click on the help button here”.* The program did not proceed to the next trial until the correct picture was selected.

Below the 4-picture array were two buttons, labeled with “help” and “talk” respectively. The “talk” button was provided so that children could listen to a target word again any time during training. This was to reduce memory load in learning new words.

If the “help” button was clicked, a visual symbol appeared on the top of the target picture. The examiner would then say “*That is the right picture. Can you click on that*?” Once the target picture was clicked, it moved automatically to the robot, prompting the robot to repeat the target word once; the program then moved to the next trial.

One point was given for each trial where a correct answer was provided on the very first attempt, even if the child had to click on the “talk” button to listen to the target word again. No point was given if the target picture was selected after more than one attempt by the child or if the “help” button was used.

Correct responses were reinforced by a cartoon figure heading a football alongside a bar: the amount the ball moved was determined by the speed of the child’s response; fast responses were rewarded with the ball being headed over the bar, to be added to the child’s store of icons. To retain interest, the icons of the headed item changed occasionally from a football to other items such as hamburgers or hedgehogs.

### Nonverbal paired associate learning task

**Figure 2:**
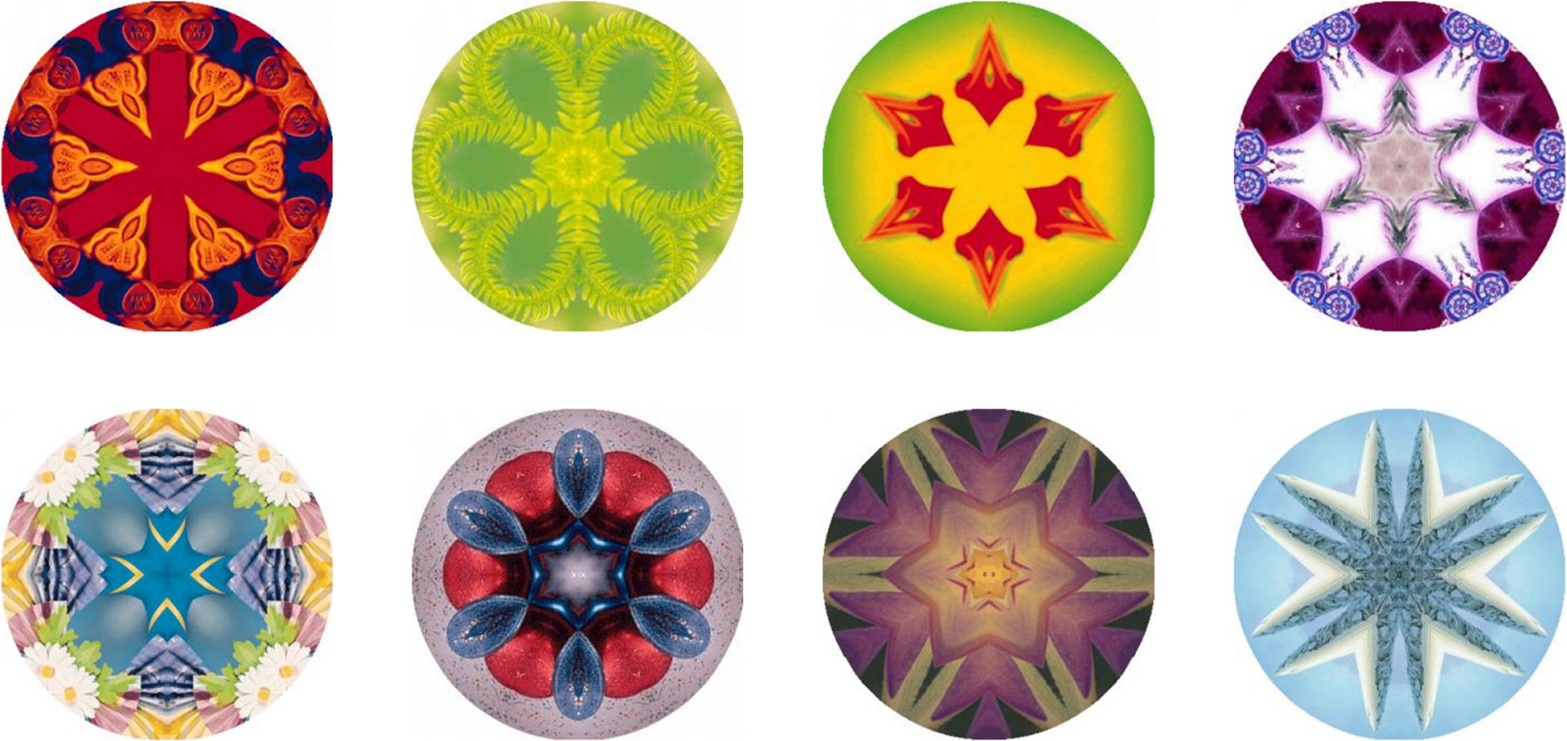
**Visual stimuli for nonverbal paired-associate learning.**

The same training program was used for the learning of sound and visual stimuli pairs, except that the animal pictures were replaced with eight visual patterns (see Figure 2), and the spoken words were replaced with complex non-speech sounds. Both visual patterns and complex sounds were devised to be meaningless and difficult to verbalise. Each sound consisted of five repetitions of a 300-ms long waveform. Each training session comprised three blocks of the eight sound and visual stimuli pairs, for a total of 24 trials. The training procedure and scoring of the paired associate learning task were the same as in the vocabulary training task.

### Word span task

In addition to the standardized tests used to identify SLI, a computerized word span task was given as an additional measure of short-term verbal memory that had not been used as part of the selection criteria (Hsu and Bishop 2014b). The child was shown a computer screen depicting a horizontal line of numbered fishing nets. The task was to select named pictures to be placed in the nets in the correct order. Children heard the spoken names and then saw an array of pictures. They were asked to click on the pictures in the same order as they had heard the words. Each click moved the picture to the next fishing net. The initial list length was three, which increased by one picture each time the list was recalled correctly in the right order. If an incorrect response was given, a second attempt at the same list length was allowed. The program stopped automatically when two successive trials were failed at a given list level. The dependent measure was word span, i.e., the longest list length at which the child gave at least one correct response. If both trials were failed at list length three, word span was recorded as two. The word lists were composed of common monosyllabic nouns.

### Data access

Stimuli, raw data and analysis programs for this paper are available on Open Science Framework at the following links: Stimuli for paired-associate training: osf.io/6pe5s. Main dataset in SPSS and SPSS script: osf.io/ujyph. R analyses using Wilcox robust methods: osf.io/igq5s.

## Results

### Learning tasks

**Figure 3:**
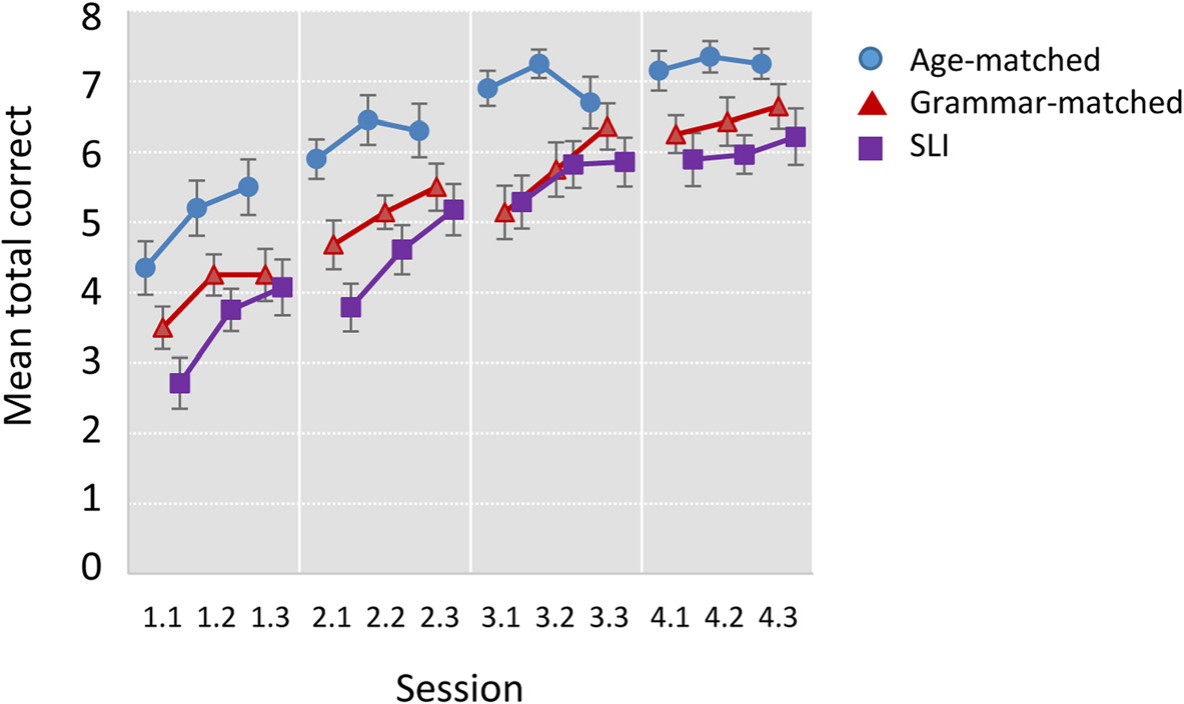
**Mean total correct by session and block for vocabulary learning task.** Error bars show standard errors.

**Figure 4:**
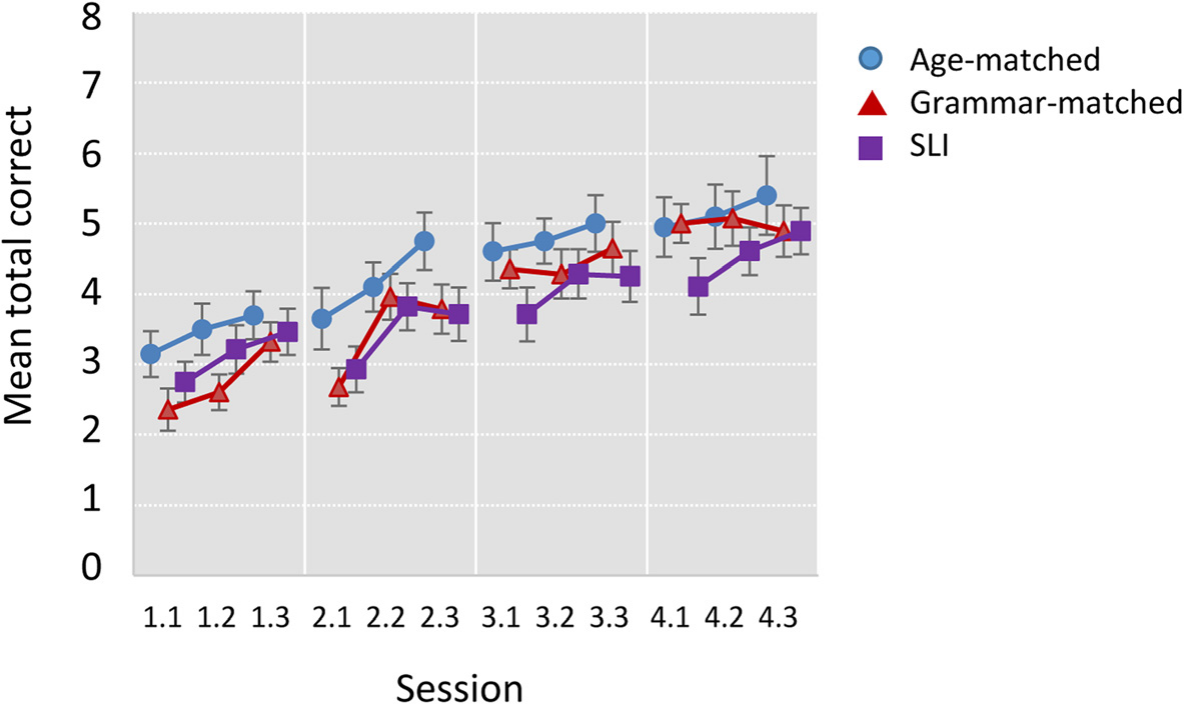
**Mean total correct by session and block for nonverbal paired-associate learning task.** Error bars show standard errors.

Figure 3 shows the mean accuracy for each block across the four training days in the SLI, Grammar-matched and Age-matched groups, and Figure 4 shows corresponding scores on nonverbal paired-associate learning. The plots indicate that, for all three groups, accuracy increases for both tasks across the four training days. The distribution of scores for the vocabulary task did not follow the normal distribution, with ceiling effects evident in the last three blocks of training. Given the violation of the normality assumption, a re-sampling method, bootstrap, was used to test for differences between groups and conditions (Wilcox 2012). The bootstrap procedure does not assume normality but instead uses the data at hand to estimate the sampling distribution of key statistics. The original data set is taken as the population from which random samples are repeatedly drawn (*bootstrap sample*) with replacement. Each of the bootstrap samples provides an estimate of the parameter of interest (e.g., mean) and relevant statistics (e.g., standard deviation), and these values are then aggregated into a bootstrap sampling distribution. This process is repeated a large number of times (default = 1000 times) to provide the required information on the variability of the estimator.

A bootstrap-t method for a three way between-within-within design was conducted using the R function *bwwtrimbt* (Wilcox 2012). There were three levels of a between-subjects factor, Group (SLI, Grammar-Matched, Age-Matched), two levels of the within-subject factor of Task (vocabulary vs nonverbal learning) and four levels of the within-subjects factor of Session (computed as total score for all three blocks for each of four training sessions). The number of bootstrap repetitions was set to 1000 with alpha level of .05. Means were trimmed by 5%.

We found significant effects of Group (p = .013), Task (p < .001) and Session (p < .001), and a significant interaction between Group and Task (p = .045), and between Task and Session (p = .013). Nonsignificant effects were found for interactions between Group and Session (p = .585) and for the three-way interaction between Group, Task and Session (p = .340). These nonsignificant effects indicate that while there is substantial learning across sessions for both tasks, the rate of learning is comparable across all groups in both tasks.

**Figure 5:**
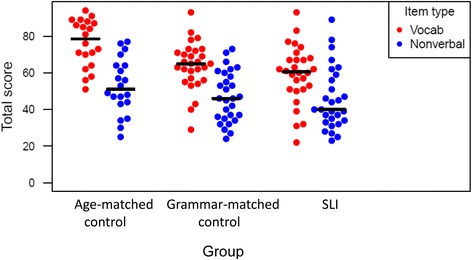
**Interaction between group and task illustrated with individual data for total scores on vocabulary and nonverbal paired-associate task.**

Post hoc tests were used with each task to explore further the interaction between group and task, which is shown in Figure 5. The *bwamcp* function (Wilcox 2012) uses the bootstrap t method to perform multiple comparisons. This was used to conduct planned pairwise comparisons for the three groups for the total scores on the two tasks (see Table 2 and Figure 5). The confidence intervals are adjusted to control the probability of at least one type I error. On the vocabulary learning task, the Age-matched group performed significantly better than the SLI and Grammar-matched groups, who did not differ from one another, whereas on the nonverbal task, the three groups did not differ significantly.

**Table 2:** **Planned comparisons between groups**

	**Mean difference,** $\widehat{\boldsymbol{\Psi}}$	**Lower 95% CI** ^**a**^	**Upper 95% CI**	**p value** ^**b**^
**Vocabulary learning**				
Grammar-match vs SLI	4.84	−5.41	15.10	0.201
Age-match vs Grammar-match	−12.60	−23.30	−1.93	0.002
Age-match vs. SLI	−17.45	−29.20	5.71	<0.001
**Nonverbal paired-associate learning**				
Grammar-match vs SLI	1.88	−9.77	13.54	0.659
Age-match vs Grammar-match	−5.98	−18.73	6.76	0.200
Age-match vs. SLI	−7.87	−21.42	5.67	0.116

Notes:

^a^ *Confidence intervals are adjusted to control familywise error rate.*

^b^ *Unadjusted p-values.*

Unfortunately, the program did not record presses of the Talk button, though testers reported these were very rare. Presses of the Help button were also rare, with a modal score of zero presses overall across all four sessions (96 trials) of the Vocabulary task. None of the age-matched group pressed Help more than twice, compared with 28 per cent of the grammar-matched group and 25 per cent of the SLI group. The maximum number of presses of Help in the Vocabulary task was 12 out of 96 trials by one child in the SLI group. The picture was very similar for the nonverbal task, with zero as the modal score, and a small tail of children in the SLI and grammar-matched groups making use of the Help button on more than two occasions. The maximum number of Help presses across all 96 trials was 13, by a child in the SLI group. These data rule out the possibility that the age-matched group did well because they were over-using the Help button.

### Predictors of vocabulary learning

In a final analysis, we considered whether initial performance or subsequent learning on vocabulary learning could be predicted by short term memory or language skills. To achieve data reduction, a preliminary principal component analysis with Varimax rotation was conducted with raw scores from the four relevant measures, (a) nonword repetition, which can be used as an index of phonological short-term memory (Archibald 2007) (b) word span, (c) receptive vocabulary, as assessed by the BPVS-2, and (d) expressive vocabulary, as assessed by ACE Naming. Two factors were extracted with eigenvalues above .9, one with high loadings from the two vocabulary tests and one with high loadings from the two memory tests. The principal components from these factors, termed Vocabulary Factor and Memory Factor respectively, were used in subsequent regression analysis.

The first regression analysis focused on predictors of the total correct for the initial session of vocabulary training, using the full sample of 76 children. Age and raw score on Raven’s matrices were entered in the first step. The Vocabulary and Memory factors were then entered together. Finally, a term coding the distinction between typically-developing and language-impaired groups was entered to check whether diagnostic category accounted for any further variance. A failure to explain further variance at this step would indicate that the differences in session 1 scores between typically-developing and SLI children were fully explained by the other variables entered into the regression.

Correlations between variables are shown in Table 3 and regression coefficients in Table 4. Age and Raven’s Matrices did not explain significant variance in Session 1 scores. (Note, however, that the failure to find an age effect could be an artefact of the specific design of the study, where age was correlated with language-impairment status). Inclusion of the Memory and Vocabulary factors accounted for significant additional variance, and examination of the beta coefficients indicated that both were significant independent predictors. Inclusion of the group contrast (typical development vs SLI) in the final step did not explain additional variance. In effect, this indicates that we completely accounted for the variance due to group when the memory and vocabulary factors were entered at the prior step of the analysis.

**Table 3:** **Correlations between age, raw cognitive/language measures and total scores for sessions 1 and 4; whole sample, N = 76**

**Variable**	**Age**	**Raven’s**	**Vocabulary**	**Memory**	**Group**	**Session 1 total**	**Session 4 total**
Age (yr)		.70**	.52**	.10	-.41**	.18	.02
Raven’s			.52**	.31**	-.19	.27*	.20
Vocabulary				.01	.20	.33**	.42**
Memory					.33**	.34**	.10
Group						.28*	.25*
Session 1 total							.41**
Mean	7.65	22.2	−0.03	0.07	0.63	12.26	19.50
SD	1.74	5.98	1.05	1.01	0.49	4.76	4.42

*p < .05; **p < .01.

**Table 4:** **Hierarchical regression analysis predicting total correct on training session 1; whole sample, N = 76**

	**ß**	**t**	**R** ^**2**^	**ΔR** ^**2**^	**p**
**Step 1**			.273	.075	.059
Age	-.03	−0.19			.851
Raven’s	.29	1.86			.067
**Step 2**			.472	.148	.002
Age	-.06	−0.39			.699
Raven’s	.04	0.23			.819
Vocabulary	.34	2.65			.010
Memory	.33	2.91			.005
**Step 3**			.490	.017	.209
Age	.07	0.37			.710
Raven’s	.08	0.49			.623
Vocabulary	.21	1.28			.203
Memory	.24	1.77			.081
TD vs SLI	.20	1.27			.209

A second analysis was conducted on scores from the final session. This was parallel to the first regression analysis, except that session 1 total score was added as a predictor at the second step prior to entering the Vocabulary and Memory factors (third step); thus this analysis identifies predictors of learning after taking into account performance in the first session. Results are shown in Table 5. Age did not account for significant variance, but Raven’s Matrices did. Inclusion of the session 1 total score explained an additional 14% of variance, and inclusion of the Memory and Vocabulary factors together explained a further 12%. This time, however, it was the Vocabulary factor that was responsible for the improved fit: Memory did not make a significant contribution to the model. Once again, inclusion of the group term (typical development vs. SLI) did not account for additional variance, indicating that any impact of group was carried by the variables entered at the previous steps.

**Table 5:** **Hierarchical regression analysis predicting total correct on training session 4; whole sample, N = 76**

	**ß**	**t**	**R** ^**2**^	**ΔR** ^**2**^	**p**
**Step 1**			.066	.066	.082
Age	-.23	−1.47			.147
Raven’s	.36	2.72			.026
**Step 2**			.204	.137	.001
Age	-.22	-1.50			.138
Raven’s	.25	1.63			.106
Session 1 total	.39	3.53			.001
**Step 3**			.327	.123	.003
Age	-.36	−2.50			.015
Raven’s	.15	0.96			.342
Session 1 total	.30	2.67			.009
Vocabulary	.43	3.45			.001
Memory	-.01	−0.09			.930
**Step 4**			.330	.003	.559
Age	.41	−2.40			.019
Raven’s	.13	0.80			.426
Session 1 total	.31	2.71			.008
Vocabulary	.49	3.13			.003
Memory	.03	0.21			.833
TD vs SLI	-.09	−0.59			.559

## Discussion

At first glance, the pattern of results we obtained may seem incompatible with the procedural deficit hypothesis, because we found that learning of new vocabulary was impaired in children with SLI. Our results are in broad agreement with a recent meta-analysis (Kan and Windsor 2010), which concluded that children with language impairment are impaired in novel word learning relative to age-matched controls, but perform at a similar level to language-matched controls. However, when we look at the pattern of results, we find evidence for preserved declarative learning in SLI.

Relative to age-matched controls, children with SLI learned fewer words from the first test block, after exposure to two instances of the name-picture pairings they had to learn. This is consistent with evidence reviewed by Lum and Conti-Ramsden (2013). In subsequent blocks, however, although their scores remained below their age peers, children with SLI made similar gains from session to session, just like younger children matched on grammatical comprehension level. In effect, the SLI deficit was completely accounted for by a difference in the intercept of the learning function, but there was no difference in the slope. Thus rate of learning of new associations and consolidation of declarative memory from one test session to the next were unimpaired in children with SLI. Furthermore, on a nonverbal paired-associate learning task using the same format, all three groups of children performed at the same level.

Following predictions by Lum and Conti-Ramsden (2013), we had anticipated that initial performance and subsequent learning might be predicted by short-term memory for nonwords or words. Only the first part of this prediction was confirmed. A memory measure, based on nonword repetition and word span, predicted performance on the first learning session, but it did not predict subsequent learning. A measure of vocabulary (based on BPVS-2 and ACE Naming), however, also predicted initial learning, and was also a predictor of subsequent gain in score between sessions 1 and 4.

It is possible that a stronger contribution from short-term memory might have been seen if we had used a learning task that required more detailed processing of speech sound information. Because we used a recognition format, with words that were highly distinctive, the child did not need to encode a detailed and accurate phonological form. Furthermore, the task was designed to minimize memory demands by allowing the child to hear a repetition of the test word if requested. With hindsight, it would have been informative to ask children to name the animals at the end of the training to obtain a measure of the precision of their phonological representations. A further point to note is that our learning task used trial-by-trial feedback. In other contexts, basal ganglia systems have been shown to be important in feedback-based learning (Seger 2008). If these systems are deficient in SLI, then this could affect task performance. To obtain optimal performance from children with SLI it might be more effective to devise a task that did not use feedback, but relied solely on incidental learning.

The finding that prior vocabulary knowledge predicted both initial learning and subsequent improvement is consistent with other studies of new vocabulary learning (Gray 2004). It could be argued that this is an unsurprising result: whatever factor helped some children to learn vocabulary in everyday life will also affect their learning on this task, and so in effect we are simply seeing two measures of the same underlying skill. There are, however, additional ways in which there could be a direct causal link between pre-existing vocacbulary and subsequent word learning. For instance, children who already possess a rich lexicon may benefit by being able to incorporate new words in a semantic network.

It was noteworthy that children with SLI were not impaired in the nonverbal paired-associate learning task, but in interpreting this result we need to also consider that there was no difference between the two control groups on this task, despite an age difference of nearly 3 years between them. This was unexpected, given that most cognitive tasks show some age progression, raising the question of whether the task was just too difficult and led to random responding. However, this does not seem to be the case. As is evident from the individual datapoints in Figure 5, the range of scores was not dissimilar for the two tasks, but the oldest control group showed a substantial advantage for the verbal task over the nonverbal task, more so than the other groups. This suggests that when language is not involved, paired-associate learning may show little change in early childhood.

## Conclusions

Overall, these results offer further support for the procedural deficit hypothesis, demonstrating that declarative memory is relatively intact in children with SLI. We showed that they have normal ability to form associations between nonverbal auditory and visual stimuli, and to remember these over time. When novel verbal stimuli were used, initial learning was impaired relative to that of age-matched controls, and the gap in performance persisted over subsequent sessions. However, rate of learning was normal and performance overall was comparable to that of younger typically-developing children whose grammatical comprehension and procedural skills were at a similar level. These results are encouraging in demonstrating an area of relative strength in children with SLI that might be exploited in intervention.
